# Premovement high‐alpha power is modulated by previous movement errors: Indirect evidence to endorse high‐alpha power as a marker of resource allocation during motor programming

**DOI:** 10.1111/psyp.12414

**Published:** 2015-02-12

**Authors:** Andrew Cooke, Germano Gallicchio, Maria Kavussanu, Adrian Willoughby, David McIntyre, Christopher Ring

**Affiliations:** ^1^ School of Sport, Health & Exercise SciencesBangor UniversityBangorUK; ^2^ School of Sport, Exercise & Rehabilitation SciencesUniversity of BirminghamBirminghamUK

**Keywords:** EEG/ERP, Error processing, Motor control, Golf, Reinvestment theory

## Abstract

Previous electroencephalographic studies have identified premovement high‐alpha power as a predictor of movement accuracy; less frontal‐central high‐alpha power is associated with accurate movements (e.g., holed golf putts), and could reflect more cognitive resources being allocated to response programming. The present experiment tested this interpretation. Ten expert and ten novice golfers completed 120 putts while high‐alpha power was recorded and analyzed as a function of whether the previous putt was holed (i.e., a correct response) or missed (i.e., an error). Existing evidence indicates that more resources are allocated to response programming following errors. We observed less premovement high‐alpha power following errors, especially in experts. Our findings provide indirect evidence that high‐alpha power is an inverse marker of the amount of resources allocated to motor response programming.

Electroencephalography (EEG) can be used to investigate the cortical correlates of successful motor performance (for reviews, see Cooke, 2013; Hatfield, Haulfler, Hung, & Spalding, 2004). Recent breakthroughs in this field have centered on the high‐alpha power frequency band (10–12 Hz). For instance, golf‐putting studies have revealed less high‐alpha power in the final seconds before and during movement in experts compared to novices and on trials where the putt was holed compared to when it was missed (Babiloni et al., 2008; Cooke et al., 2014). Given the reported inverse relationship between alpha power and cortical activity (e.g., Pfurtscheller, 1992), these findings have been interpreted to indicate that more cortical resources are allocated to the accurate programming of movement parameters, such as direction and force, during successful trials (Cooke et al., 2014). However, this interpretation does not sit well with models of motor automaticity (e.g., Hatfield & Hillman, 2001), which argue that optimal performances are characterized by the recruitment of fewer resources, not more (i.e., an economy of effort). In light of this counterargument, this report was designed to revisit the assertion that reduced premovement high‐alpha power reflects more resources being allocated to the programming of golf putts.

To test this interpretation, we investigated the extent to which preparatory high‐alpha power is influenced by the previous trial. When performing multiple repetitions of a motor task, the outcome of the previous trial is considered to have a profound influence on the amount of resources allocated to programming the next movement, with more resources allocated if the previous trial contained an error. For instance, Lam, Masters, and Maxwell (2010) showed that probe reaction times to a tone presented during preparation for golf putts were longer when the previous putt was missed compared to when it was holed. Elongated probe reaction times following errors were interpreted to indicate that more resources were devoted to motor programming of the subsequent movement. For example, a golfer whose previous putt missed to the left of the target would devote resources to reprogram/parameterize the motor commands ahead of the next trial.

This assertion is in line with reinvestment theory (Masters & Maxwell, 2008), a model of motor performance that posits that performance errors prompt individuals to reinvest cognitive resources in an attempt to take conscious control of their movements. Experts are assumed to be especially susceptible to reinvestment because they are more sensitive to errors than novices, and because they have a greater bank of performance‐relevant resources to allocate to the task (Beilock & Carr, 2001; Lam et al., 2010). For example, if a professional golfer misses a 5‐foot putt, they may call upon a detailed knowledge of how to adjust their movement to prevent further errors from occurring. In contrast, a novice golfer is likely to possess much less knowledge of how their movement can be changed.

In accord with this theorizing, we formulated two hypotheses. First, we expected lower high‐alpha power in the final seconds preceding trials when the previous putt was missed compared to when it was holed. Second, we predicted that this effect would be moderated by ability, such that high‐alpha power on trials following errors would be suppressed to a greater extent in experts than novices. Our hypotheses were tested using new analyses performed on an existing dataset (see Cooke et al., 2014).

## Method

### Participants

Ten expert (*M* age = 20.90, *SD* = 0.74 years) and ten novice (*M* age = 19.00, *SD* = 0.66 years) right‐handed male golfers volunteered to participate. The experts had a mean of 11.25 (*SD* = 3.78) years of golf experience and a golf handicap < 5. The novices had a mean of 1.85 (*SD* = 2.49) years of golf experience and no formal golf handicap. All participants provided informed consent. The protocol was approved by the local research ethics committee.

### Task

Participants used a standard length (90 cm) golf putter to putt regular‐size (diameter 4.7 cm) golf balls toward a hole on an artificial putting mat from a distance of 2.4 m. The hole had a diameter of 10.8 cm (i.e., standard size) for novices and 5.4 cm for experts. This distance and two hole sizes yielded a success rate of 66%, thus ensuring a sufficient and similar number of successful and unsuccessful trials for statistical comparison.

### Design

We adopted a mixed multifactorial design, with group (novice, expert) as a between‐subjects factor, and previous trial outcome (previous putt holed, previous putt missed), and epoch (−4 to −3 s, −3 to −2 s, −2 to −1 s, −1 s to 0 s, 0 s to +1 s) as within‐subjects factors. Epoch refers to the time windows around movement during which cortical activity was assessed.

### Measures

EEG activity was recorded from 16 silver/silver chloride electrodes on the scalp (Fp1, Fp2, F4, Fz, F3, T7, C3, Cz, C4, T8, P4, Pz, P3, O1, Oz, O2) positioned using the 10‐20 system (Jasper, 1958). Electrodes were also placed at the left and right mastoids, to permit offline referencing. Signals were amplified and digitized at 512 Hz with 24‐bit resolution (ActiveTwo, BioSemi) using Actiview software (BioSemi).

### Procedure

Participants attended a 2‐h testing session. Following instrumentation, participants were instructed to try to get all putts “ideally in the hole, but if unsuccessful, to make them finish as close to the hole as possible.” Next, they performed 20 familiarization putts to become accustomed to the putting surface and to putting while instrumented for EEG recordings. Participants then performed 120 test putts in two 60‐putt blocks, which were averaged.^1^ The interval between putts ranged approximately from 17–25 s. After each putt, the outcome was recorded, and then the ball was replaced at the start position by the experimenter. Participants were debriefed and thanked when the session was complete.

### Data Reduction

Individual trials within the continuous EEG recordings were identified using an optical sensor (S51‐PA 2‐C10PK, Datasensor) and a microphone (NT1, Rode) whose signals were recorded using Actiview (BioSemi) and Spike2 (Cambridge Electronic Design) software. Once trials were identified, the signals were filtered (1–50 Hz) and referenced to the average mastoid, and artifacts including eye movements and blinks were identified and removed using independent component analyses (Delorme & Makeig, 2004) and the ADJUST algorithm (Mognon, Jovicich, Bruzzone, & Buiatti, 2010), as reported elsewhere (Cooke et al., 2014). An average of 114 (*SD* = 10.61) trials per participant were retained. These artifact‐free data were then averaged in successive 1‐s epochs from 4 s before until 1 s after the initiation of putts, relative to a −4 s to −3 s baseline, and high‐alpha power (10–12 Hz) was computed (fast Fourier transform, 1 Hz bins, Hanning window taper). Importantly, 2 (Group) × 2 (Previous Outcome) analyses of variance (ANOVAs) performed prior to baseline removal revealed no high‐alpha power main or interaction effects during the −4 s to −3 s baseline. This confirmed that the baseline was not differentially influenced by any of the factors in our experiment (i.e., was neutral).

### Statistical Analyses

**Figure 1:**
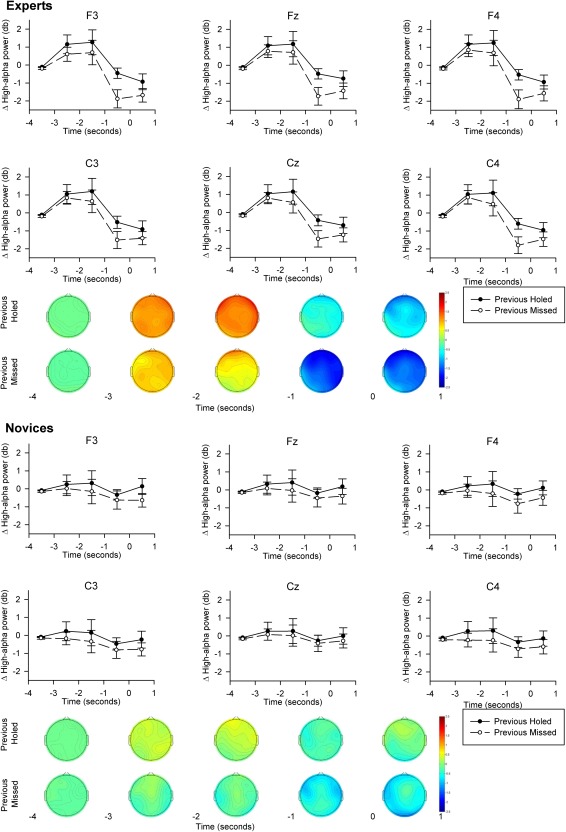
Line plots and topographic scalp maps to depict effects of previous outcome on high‐alpha power in experts and novices. Zero on the horizontal (time) axis indicates the initiation of putts. Error bars indicate standard error of the means.

High‐alpha power at each site was subjected to a 2 (Group) × 2 (Previous Outcome) × 5 (Epoch) ANOVA. Significant effects were probed by *t* tests and polynomial trend analyses, while interactions involving group were probed by 2 (Previous Outcome) × 5 (Epoch) ANOVAs conducted separately for experts and novices. The results of multivariate tests are reported below. Multivariate analyses do not make the assumption of sphericity so corrections to the reported degrees of freedom were not necessary (Vasey & Thayer, 1987). For brevity, only the results of the Fz, F3, F4, Cz, C3, and C4 electrodes are presented. Topographic analyses revealed that these electrode sites were largely representative of the others while capturing the strongest effects (Figure 1)

## Results

Our results are illustrated in Figure 1. The 2 (Group) × 2 (Previous Outcome) × 5 (Epoch) ANOVAs revealed main effects of previous outcome, *F*s(1,18) = 4.67–6.72, *p*s < .05, $\eta_{p}^{2}$s = .21–.27, and epoch, *F*s(4,15) = 3.17–3.80, *p*s < .05, $\eta_{p}^{2}$s = .46–.50, at F3, F4, Fz, C3, and C4 sites. Previous Outcome × Epoch interaction effects were also observed at F3, Fz, and F4, *F*s(4,15) = 3.15–3.67, *p*s < .05, $\eta_{p}^{2}$s = .46–.50. In brief, high‐alpha power displayed a linear polynomial trend that was stronger, while high‐alpha power was less, on trials that followed a missed putt (i.e., an error) compared to those that followed a holed putt.

Finally, and most importantly, these effects were superseded by Group × Previous Outcome × Epoch interaction effects at C3, Cz, F3, and Fz, *F*s(4,5) = 3.05–4.16, *p*s < .05, $\eta_{p}^{2}$s = .45–53. Subsequent 2 (Previous Outcome) × 5 (Epoch) ANOVAs, performed separately for experts and novices, revealed that the Previous Outcome × Epoch linear trends only reached significance in experts (at Fz and F3), *F*s(1,9) = 5.24–6.13, *p*s < .05, $\eta_{p}^{2}$s = .37–.41. This was underscored by *t* tests, which indicated that experts produced less high‐alpha power in the −3 s to −2 s (at F3) and −1 s to 0 s (at all sites) epochs when the previous putt was missed compared to when it was holed, *t*s(9) = 2.75–3.13, *p*s < .05, *d*s = .66–.94.

## Discussion

This report aimed to shed light on the role of high‐alpha power during the final moments of preparation for a motor task. Specifically, it revisited the previously reported assertion that less high‐alpha power reflects more resources being allocated to motor programming during preparation for golf putts (Cooke et al., 2014). This was important because recent research has associated lower premovement high‐alpha power with optimal performance (Babiloni et al., 2008; Cooke et al., 2014), a state that was previously thought to be characterized by the mobilization of fewer, not more, programming resources (for review, see Hatfield & Hillman, 2001).

Our first hypothesis was that high‐alpha power would be lower in the final seconds preceding trials where the previous putt was missed, compared to when it was holed. This hypothesis was supported. Lam and colleagues (2010) reported that golfers allocated more resources to response programming when their previous putt was missed. Our finding that high‐alpha power was also reduced when the previous putt was missed thus offers indirect support to Cooke et al.'s (2014) view that reduced high‐alpha power reflects an increase in the amount of resources allocated to response programming during a putting task.

Our second hypothesis was that the effect of previous outcome on high‐alpha power would be moderated by ability, with high‐alpha power in the seconds preceding putts that followed a miss being suppressed to a greater extent by experts than by novices. This hypothesis was derived from reinvestment theory (Masters & Maxwell, 2008), which argues that experts have more resources than novices to devote to error monitoring and correction (Beilock & Carr, 2001; Lam et al., 2010). In line with expectations, the observed Group × Previous Outcome × Epoch interactions confirmed that previous outcome differences in high‐alpha power were stronger in experts than in novices (Figure 1). To our knowledge, this represents the first objective neuroscientific evidence to support this key prediction of reinvestment theory.

## Limitations and Future Directions

While our results imply that high‐alpha power could reflect the amount of resources allocated to a task, they offer no insight into how resources are utilized. By conducting EEG coherence analyses, researchers could determine the extent to which performers engage in specific elements of preparation such as verbal‐analytic versus visuospatial processing (e.g., Zhu, Poolton, Wilson, Maxwell, & Masters, 2011). Increased verbal‐analytic processing can impair performance (Zhu et al., 2011), so it is important that increased resources are allocated to appropriate elements of preparation, for performance benefits to be realized. Future research could also employ a more direct test of whether high‐alpha power reflects the amount of resources allocated to a task by manipulating response programming demands, such as comparing high‐alpha power for easy versus difficult putts.

## Conclusion

Our results provide indirect evidence that high‐alpha power represents an inverse marker of the amount of resources devoted to motor response programming. They also provide support for reinvestment theory's prediction that expert performers are especially likely to increase the amount of cognitive resources devoted to motor planning when there is a need to correct for previous errors. Together with previous golf‐putting research (i.e., Babiloni et al., 2008; Cooke et al., 2014), these results can be interpreted to challenge the popular view that the mobilization of fewer motor programming resources characterize sporting excellence. Instead, they suggest that, at least for the skill of golf putting, players should make an effort to increase the amount of resources they allocate to programming key movement parameters, to achieve putting success.

## Footnotes

1 The two blocks of 60 putts represented low‐ and high‐pressure conditions, but they have been averaged in this report because the pressure manipulation had no impact on cortical activity (see Cooke et al., 2014, for a full description of this pressure manipulation).
